# Establishment of a prognostic nomogram based on the clinical and inflammatory parameters as well as acute radiation enteritis for patients with cervical cancer receiving radiotherapy

**DOI:** 10.3389/fonc.2024.1453837

**Published:** 2024-11-29

**Authors:** Jing Hu, Qianjin Shi, Xiaoqin Gong, Tao You, Chunhua Dai, Fei Chen

**Affiliations:** ^1^ Department of Oncology, Affiliated Hospital of Jiangsu University, Zhenjiang, Jiangsu, China; ^2^ Department of Oncology, Siyang Hospital, Suqian, Jiangsu, China

**Keywords:** cervical cancer, radiotherapy, acute radiation enteritis, prognosis, nomogram

## Abstract

**Objective:**

Acute radiation enteritis is one of the most common complications of radiotherapy for patients with cervical cancer. This study aims to investigate the effect of acute radiation enteritis on the prognosis of patients with cervical cancer receiving radiotherapy and to establish a nomogram predicting the patients’ overall survival (OS).

**Methods:**

The clinical data of 288 patients with cervical cancer who were admitted to our department from 2014 to 2020 were retrospectively analyzed, and the survival of patients were followed up. The Kaplan-Meier method was used to calculate the survival rate and for univariate analysis, and the Cox regression model was used for multivariate prognostic analysis. A nomogram survival prediction model was established based on independent risk factors, and the concordance index (C-index), receiver operating characteristic (ROC) curve and calibration curve were used to evaluate the predictive accuracy of the model. The clinical applicability of the model was assessed by the decision curve. External validation of the nomogram prediction model was performed in 74 patients admitted to our hospital from 2020 to 2021.

**Results:**

60 patients (20.8%) developed grade 2 or higher acute radiation enteritis. The 1-, 3-, and 5-year OS rates were 94.4%, 80.9%, and 77.4%, respectively. Multivariate Cox regression analysis showed that: Age ≥ 60 years, diabetes/hypertension, anemia, FIGO stage III-IV, poor differentiation, pelvic lymph node metastasis, NLR ≥ 2.54 and grade 2 or higher acute radiation enteritis were independent risk factors for OS in cervical cancer patients undergoing radiotherapy (*P* < 0.05). The C-index of OS nomogram model was 0.815 (95% CI: 0.766-0.864). The AUC of 3-year and 5-year OS were 0.849 (95%CI: 0.789-0.909) and 0.840 (95%CI: 0.782-0.899), respectively. The AUC value of 3-year OS in the external validation set was 0.779 (95%CI: 0.635-0.922). The calibration curve showed that the model was well calibrated, and the decision curve verified the clinical applicability of the constructed nomogram.

**Conclusion:**

This study established an accurate predicting nomogram based on independent prognostic factors in cervical cancer patients receiving radiotherapy, and patients with grade 2 or higher acute radiation enteritis should be paid more attention to in clinical practice.

## 1 Introduction

Cervical cancer is the most common gynecological malignant tumor in China. Radiotherapy is one of the main treatment methods for cervical cancer, and the vast majority of patients require radiotherapy, which can improve the survival rate, but also lead to radiotherapy complications (1–3). With the improvement of radiotherapy technology, the number of patients receiving intensity-modulated radiotherapy (IMRT) is increasing. Compared with three-dimensional conformal radiotherapy (3D-CRT), IMRT can reduce the dose to the intestines, thereby alleviating gastrointestinal toxic reactions (4–6). However, acute radiation enteritis (ARE) is one of the most common complications of radiotherapy for cervical cancer, which can seriously reduce the quality of life of patients, lead to treatment interruption, and even affect the clinical efficacy (7). Many studies have shown that a variety of factors, such as patient clinical characteristics, dosimetric factors, and treatment methods, are related to the occurrence of radiation enteritis (8, 9). However, the effect of acute radiation enteritis on the prognosis of patients with cervical cancer has not yet been studied.

In recent years, the relationship between pre-treatment inflammatory indicators and the prognosis of patients with tumors has gradually attracted attention. Inflammation plays an important role in the growth of new tumor angiogenesis and in promoting tumor metastasis (10, 11). As the first responders of inflammation, neutrophils have been increasingly recognized to participate in the tumorigenesis and progression (12, 13). Neutrophil-to-lymphocyte ratio (NLR), monocyte-to-lymphocyte ratio (MLR) and platelet-to-lymphocyte ratio (PLR), as inflammatory indicators, are considered to be closely related to the severity and prognosis of many diseases (14–16). However, there are few studies on whether NLR, MLR, and PLR can be used as predictors of radiotherapy efficacy for cervical cancer patients.

Existing studies have identified numerous factors affecting the prognosis of cervical cancer patients (17–19), yet the specific impact of acute radiation enteritis on the prognosis of these patients remains unclear and under-researched. Therefore, this study aims to collect clinical data from cervical cancer patients and follow up on their survival status, with a particular focus on analyzing the impact of acute radiation enteritis on the prognosis of cervical cancer patients.

## 2 Materials and methods

### 2.1 Clinical data

A retrospective analysis was conducted on cervical cancer patients who received radiotherapy in our department from 2014 to 2020. Patients with distant metastasis (stage IVB), a history of other malignancies, or abnormal cardiopulmonary function were excluded, resulting in a total of 288 cases. All patients had a Karnofsky Performance Status (KPS) score of ≥70 before treatment. The patients’ ages ranged from 24 to 85 years, with a median age of 54 years. According to the Federation International of Gynecology and Obstetrics (FIGO) 2014 staging system, 79 patients were in stage IB, 137 in stage II, 68 in stage III, and 4 in stage IVA. Pathological types included 249 cases of squamous cell carcinoma, 34 cases of adenocarcinoma, and 5 cases of adenosquamous carcinoma. A total of 147 patients received definitive radiotherapy, while 141 patients received postoperative adjuvant radiotherapy. The radiotherapy methods included 3D-CRT for 134 patients and IMRT for 154 patients. This study was approved by the Ethics Committee of our hospital (Approval No. KY2021K0901).

### 2.2 Treatment methods

All the patients were fixed with a vacuum cushion in the supine position. The enhanced computed tomography (CT) scan was performed for patients using a Philips Big Bore scanner, and the slices were reconstructed with a separation of 3 mm. The scanned images were transmitted to the Eclipse 13.6 treatment planning system of our department through DICOM for clinical target volume and organs at risk (OARs) delineation. External beam radiation therapy (EBRT) was delivered in 25 fractions, with a total dose of 45-50 Gy. For patients with positive pelvic lymph nodes, an additional 10-20 Gy was administered. Patients who were inoperable or intolerant to surgery received definitive radiotherapy (combination of EBRT and brachytherapy), with a total prescribed dose of 80-85 Gy. Patients with positive surgical margins or close margins (cancerous tissue within 5mm of the margin) also received supplementary brachytherapy, with a reference point 0.5 cm below the vaginal mucosa. The chemotherapy regimen was primarily platinum-based.

### 2.3 Data collection

Data collected included patient age, comorbidity (hypertension/diabetes), nutritional status, height, weight, tumor size, histopathological type, FIGO stage, and lymph node metastasis status (LNM). Pre-radiotherapy complete blood count results were recorded to calculate the neutrophil-to-lymphocyte ratio (NLR), monocyte-to-lymphocyte ratio (MLR), and platelet-to-lymphocyte ratio (PLR). All patients were regularly screened for nutritional risk (at least once a week), and patient-generated subjective global assessment (PG-SGA) was used for nutritional assessment in patients at nutritional risk to determine the presence of malnutrition (20). Adverse reactions were monitored weekly during treatment, and the severity of acute radiation enteritis (including diarrhea, enterocolitis, and proctitis) was graded according to the U.S. Department of Health and Human Services (HHS) Common Terminology Criteria for Adverse Events (CTCAE) 5.0, as shown in **Table 1**.

**Table 1:** Common Terminology Criteria for Adverse Events (CTCAE) Version 5.0.

CTCAE Term	Grade 1	Grade 2	Grade 3	Grade 4	Grade 5
Diarrhea	Increase of <4 stools per day over baseline	Increase of 4 - 6 stools per day over baseline	Increase of ≥7 stools per day over baseline; hospitalization indicated	Life-threatening consequences; urgent intervention indicated	Death
Enterocolitis	Asymptomatic; clinical or diagnostic observations only; intervention not indicated	Abdominal pain; mucus or blood in stool	Severe or persistent abdominal pain; fever; ileus; peritoneal signs	Life-threatening consequences; urgent intervention indicated	Death
Proctitis	Rectal discomfort, intervention not indicated	Symptomatic (e.g., rectal discomfort, passing blood or mucus); medical intervention indicated; limiting instrumental ADL	Severe symptoms; fecal urgency or stool incontinence; limiting self-care ADL	Life-threatening consequences; urgent intervention indicated	Death

### 2.4 Patients follow-up

The follow-up period ended on December 30, 2022. Follow-up methods included inpatient or outpatient reviews and telephone follow-ups. Overall survival (OS) was defined as the time from cervical cancer diagnosis to death from any cause.

### 2.5 Statistical analysis

Statistical analysis was performed using SPSS 22.0 software. The optimal cutoff values of NLR, MLR, PLR, and each dosimetric parameter were determined using the Youden index of receiver operating characteristic (ROC) curves. NLR cut-off value of 2.54 was calculated using the ROC curves, and the cutoff values for MLR and PLR were determined using the same method. Survival rates were calculated using the Kaplan-Meier method, and univariate analysis was conducted using the Log-rank test. Multivariate prognostic analysis was performed using the Cox regression model. *P*<0.05 was considered statistically significant. Based on the results of the multivariate analysis, a nomogram prediction model was established using the rms package in Rstudio (R4.3.1). The predictive accuracy of the model was evaluated using the concordance index (C-index), ROC curves, and calibration curves, while decision curve analysis was used to assess the clinical utility of the model. External validation was performed based on 74 cases from 2020 to 2021.

## 3 Results

### 3.1 Incidence of acute radiation enteritis

Among the 288 patients in this cohort, 208 (72.2%) experienced acute radiation enteritis during or within three months after radiotherapy. According to the grading criteria for acute radiation enteritis, 80 cases (27.8%) were grade 0, 148 cases (51.4%) were grade 1, 51 cases (17.7%) were grade 2, and 9 cases (3.1%) were grade 3. There were no cases of grade 4 or 5. The incidence of grade 2 or higher acute radiation enteritis was 20.8%.

### 3.2 Status of survival of all patients

The follow-up period ranged from 6 to 106 months, with a median follow-up of 59 months. By the end of the follow-up period, 72 patients had died, and 2 patients were lost to follow-up, resulting in a follow-up rate of 99.3%. The 1-year, 3-year, and 5-year overall survival (OS) rates were 94.4%, 80.9%, and 77.4%, respectively.

### 3.3 Prognostic factors analysis

**Figure 1:**
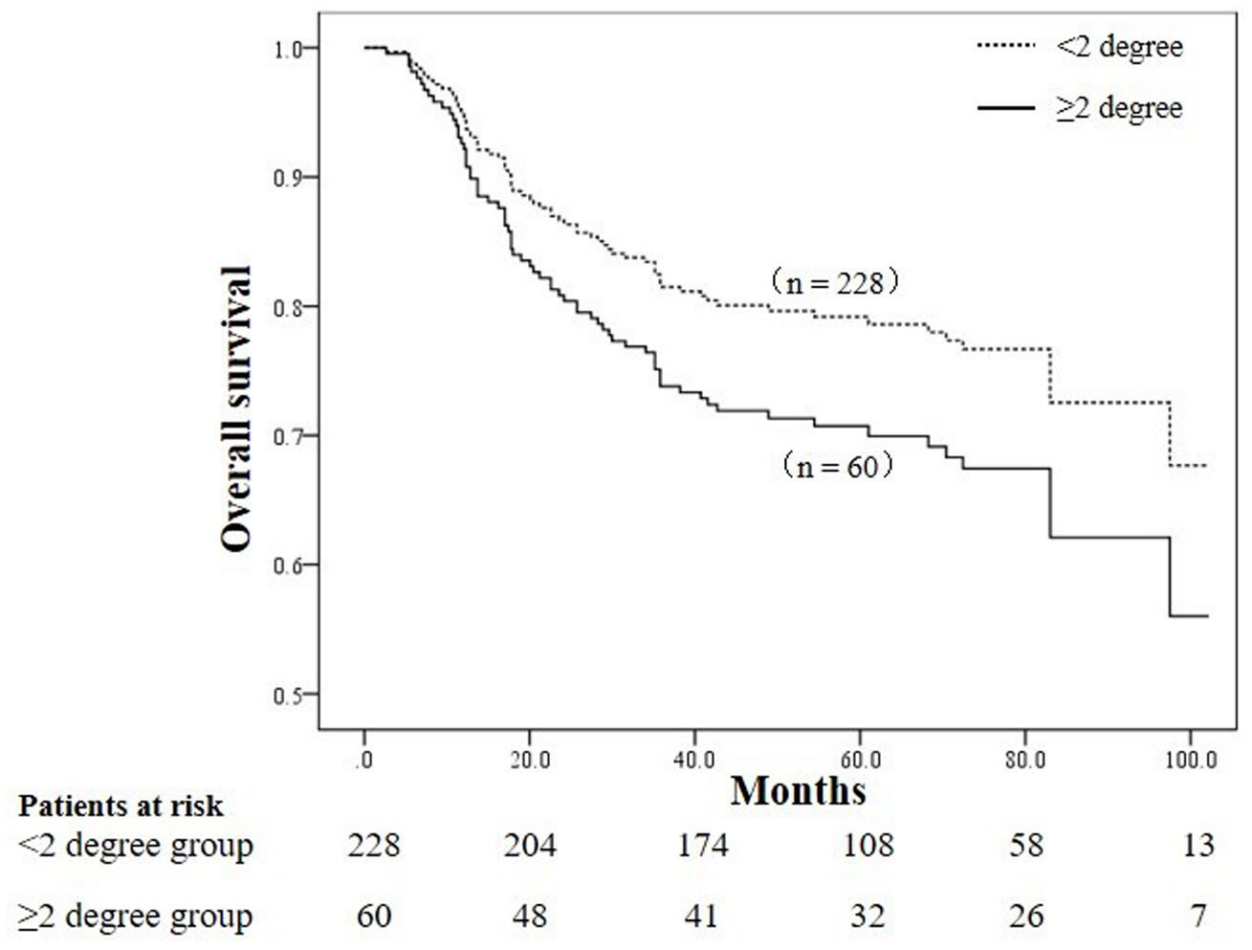
Comparison of overall survival curves between patients with grade ≥ 2 acute radiation enteritis and grade < 2 acute radiation enteritis (*P* = 0.044).

Univariate analysis indicated that age ≥60 years, comorbid diabetes/hypertension, anemia, postoperative radiotherapy, FIGO stage, differentiation degree, pelvic lymph node metastasis, receipt of brachytherapy, NLR level, MLR level, PLR level, V5, V10, V20 of the bowelbag were prognostic factors for cervical cancer patients undergoing radiotherapy, as shown in **Tables 2**, **3**. The OS of patients with grade 2 or higher acute radiation enteritis was lower than that of patients with grade 0-1 acute radiation enteritis, although the difference was not statistically significant (*P* = 0.127). Multivariate Cox regression analysis revealed that age ≥60 years (*P* < 0.001), comorbid diabetes/hypertension (*P* = 0.005), anemia (*P* < 0.001), FIGO stage III-IV (*P* = 0.028), poor differentiation (*P* < 0.001), pelvic lymph node metastasis (*P* < 0.001), NLR ≥ 2.54 (*P* = 0.048), and grade 2 or higher acute radiation enteritis (*P* = 0.044) were independent risk factors for OS in cervical cancer patients undergoing radiotherapy, as shown in **Table 4**. The OS curves stratified by the severity of acute radiation enteritis were shown in **Figure 1**, indicating that patients with grade 2 or higher acute radiation enteritis had significantly lower OS rate compared to those with less than grade 2.

**Table 2:** Univariate analysis of general clinical data on the prognosis of cervical cancer patients undergoing radiotherapy (n = 288).

Variable	Patients n	5-year OS (%)	χ^2^	*P*	Variable	Patients n	5-year OS (%)	χ^2^	*P*
Age			25.759	<0.001	FIGO Stage			57.686	<0.001
<60	181	86.2			I-II	216	86.7		
≥60	107	62.4			III-IV	72	49.7		
Comorbidity			11.597	0.001	Chemotherapy			0.105	0.746
No	195	82.1			No	74	74.3		
Yes	93	67.6			Yes	214	78.3		
Nutrition			0.643	0.423	CCRT			0.167	0.682
Well	178	79.4			No	122	75.4		
Poor	110	74.2			Yes	166	78.6		
Anemia			13.163	<0.001	RT technology			2.673	0.102
No	263	79.9			CRT	134	82.8		
Yes	25	51.3			IMRT	154	71.9		
BMI			0.001	0.973	Brachytherapy			36.581	<0.001
18.5-23.9	177	78.1			No	135	92.8		
<18.5 or ≥24	111	76.4			Yes	153	63.8		
Tumor size			2.348	0.125	NLR			4.647	0.031
<4 cm	149	81.9			<2.54	172	80.1		
≥4 cm	139	72.7			≥2.54	116	73.2		
Surgery			38.090	<0.001	MLR			8.057	0.005
No	147	63.7			<0.24	119	85.5		
Yes	141	91.6			≥0.24	169	71.7		
Differentiation			13.382	0.001	PLR			10.844	0.001
Unknown	82	73.9			<120.42	111	69.0		
Well-Moderate	134	85.5			≥120.42	177	82.7		
Poor	72	66.5							
Pelvic LNM			21.371	<0.001	ARE			0.437	0.509
No	210	84.0			No	80	78.8		
Yes	78	59.6			Yes	208	76.9		
Pathological type			1.765	0.184	Grade of ARE			2.326	0.127
Squamous	249	78.7			Grade 0-1	228	79.2		
Non-squamous	39	69.2			Grade 2-3	60	70.0		

BMI, body mass index; LNM, lymph node metastasis; FIGO, Federation International of Gynecology and Obstetrics; CCRT, concurrent chemoradiotherapy; RT technology, radiotherapy technology; NLR, Neutrophil-to-lymphocyte ratio; MLR, monocyte-to-lymphocyte ratio; PLR, platelet-to-lymphocyte ratio.

**Table 3:** Univariate analysis of intestinal dosimetric parameters on the prognosis of cervical cancer patients undergoing radiotherapy (n = 288).

Parameters (Bowelbag)	Patients n	5-year OS (%)	χ2	*P*	Parameters (Rectum)	Patients n	5-year OS (%)	χ2	*P*
V5/ml			9.090	0.003	V5/ml			0.001	0.982
< 1649.55	227	80.5			< 80.35	237	76.5		
≥ 1649.55	61	65.6			≥ 80.35	51	81.3		
V10/ml			4.768	0.029	V10/ml			0.001	0.982
< 1367.08	204	80.5			< 80.35	237	76.5		
≥ 1367.08	84	70.0			≥ 80.35	51	81.3		
V20/ml			9.370	0.002	V20/ml			0.056	0.812
< 1200.77	204	81.5			< 42.22	105	77.1		
≥ 1200.77	84	67.6			≥ 42.22	183	77.5		
V30/ml			2.875	0.090	V30/ml			0.077	0.781
< 993.87	247	78.0			< 41.74	106	77.4		
≥ 993.87	41	73.2			≥ 41.74	182	77.4		
V40/ml			3.639	0.056	V40/ml			0.163	0.687
< 356.04	106	69.4			< 84.56	266	77.0		
≥ 356.04	182	81.7			≥ 84.56	22	81.6		
V50/ml			0.236	0.627	V50/ml			2.342	0.126
< 128.62	183	76.7			< 25.18	209	78.3		
≥ 128.62	105	78.1			≥ 25.18	79	74.6		
Dmean/Gy			3.092	0.079	Dmean/Gy			0.189	0.664
< 30.28	166	71.9			< 46.67	171	77.2		
≥ 30.28	122	84.4			≥ 46.67	117	77.6		

**Table 4:** Multivariate cox regression analysis of prognostic factors in cervical cancer patients undergoing radiotherapy (n = 288).

Variable	β	SE	Wald	*P*-value	HR(95% CI)
Age	1.229	0.332	13.706	<0.001	3.417(1.783-6.549)
Comorbidity	0.741	0.267	7.720	0.005	2.099(1.244-3.541)
Anemia	1.701	0.390	19.059	<0.001	5.482(2.554-11.767)
FIGO Stage	0.666	0.303	4.827	0.028	1.947(1.075-3.529)
Differentiation			20.994	<0.001	
Poor vs. Unknown	0.404	0.352	1.317	0.251	1.498(0.751-2.986)
Poor vs. Well-Moderate	1.471	0.339	18.794	<0.001	4.353(2.239-8.466)
Pelvic LNM	1.280	0.272	22.107	<0.001	3.595(2.109-6.128)
NLR	0.590	0.298	3.926	0.048	1.804(1.006-3.232)
Grade of ARE	0.598	0.296	4.073	0.044	1.818(1.017-3.247)

FIGO, Federation International of Gynecology and Obstetrics; LNM, lymph node metastasis; NLR, Neutrophil-to-lymphocyte ratio; ARE, acute radiation enteritis.

### 3.4 Establishment and evaluation of the nomogram survival prediction model

**Figure 2:**
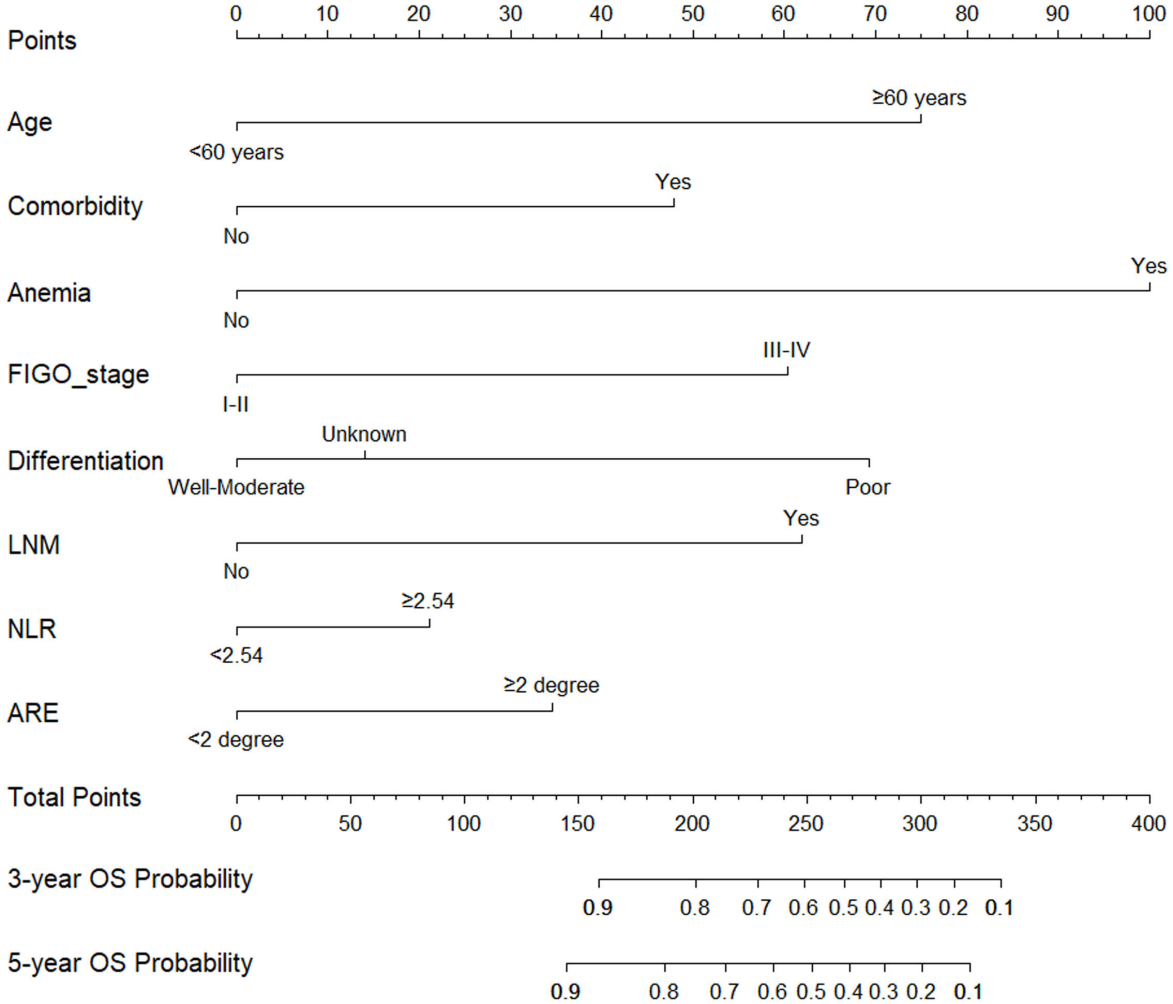
Nomogram prediction model for 3-year and 5-year overall survival in cervical cancer patients undergoing radiotherapy. Locate “<60 years” or “≥60 years” on the horizontal axis of age, draw a vertical line upward on this horizontal axis to determine point obtained for this patient, and repeat this process for other horizontal axis parameters (comorbidity, anemia, FIGO stage, differentiation, LNM, NLR, ARE), and then add the sum of all parameter points together, locate its position on the horizontal axis of the total points, and draw a vertical line down to determine the 3-year, 5-year OS probability. FIGO, federation international of gynecology and obstetrics; LNM, lymph node metastasis; NLR, neutrophil-to-lymphocyte ratio; ARE, acute radiation enteritis.

**Figure 3:**
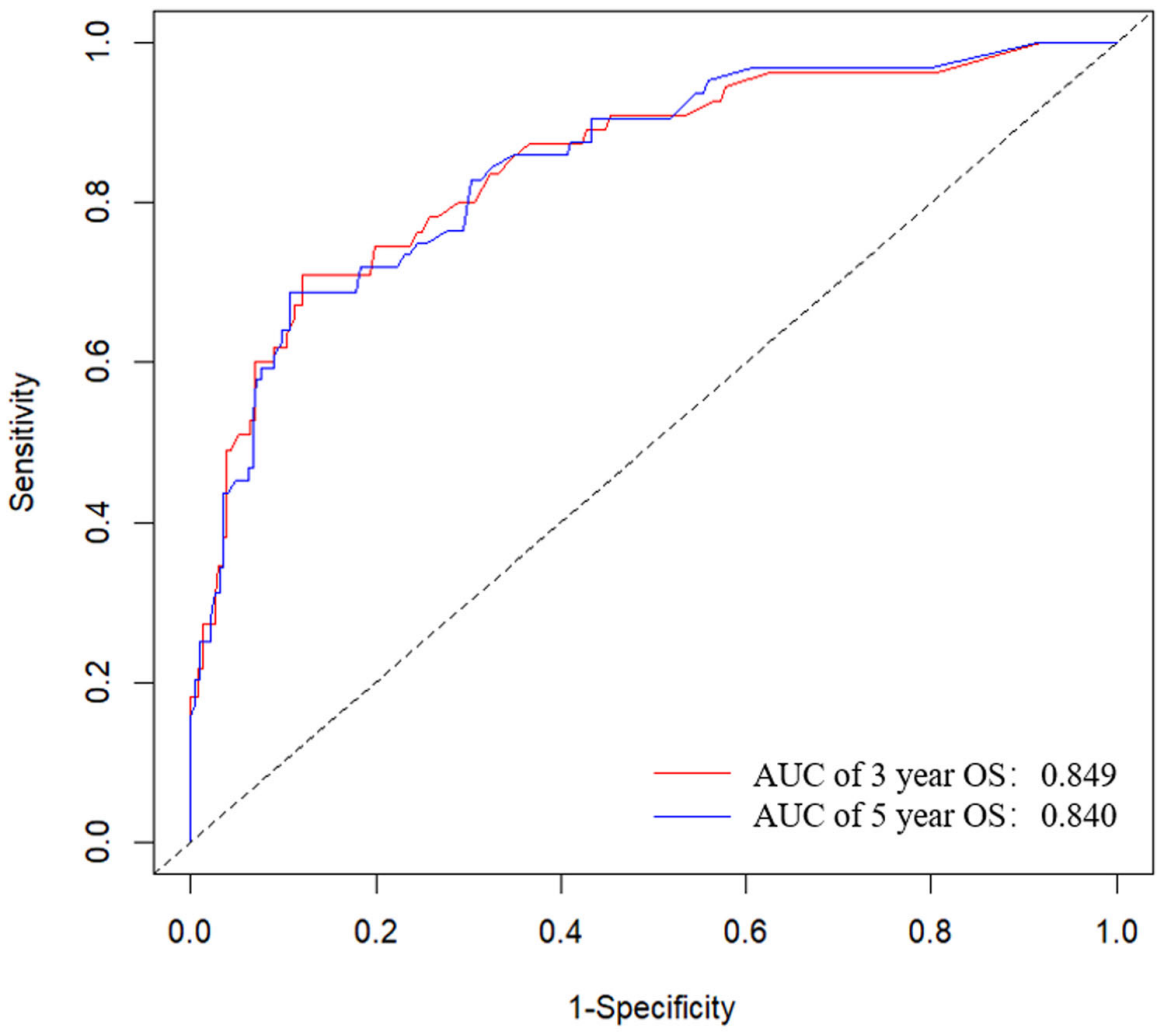
ROC curves for the nomogram prediction model of 3-year and 5-year overall survival in cervical cancer patients undergoing radiotherapy. The AUC values of 3-year and 5-year OS were 0.849(95%CI: 0.789-0.909) and 0.840 (95%CI: 0.782-0.899). ROC, receiver operating characteristic; AUC, area under the ROC curve.

**Figure 4:**
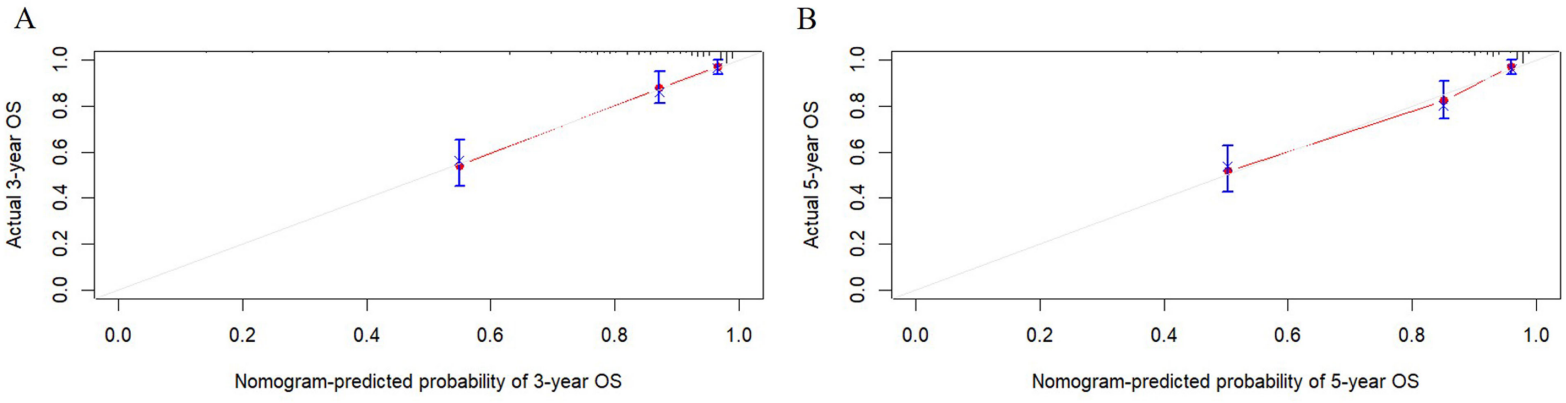
Calibration curves for the nomogram prediction model of overall survival in cervical cancer patients undergoing radiotherapy. **(A)** Calibration curve for predicting probability of the 3-year OS. **(B)** Calibration curve for predicting probability of the 5-year OS.

**Figure 5:**
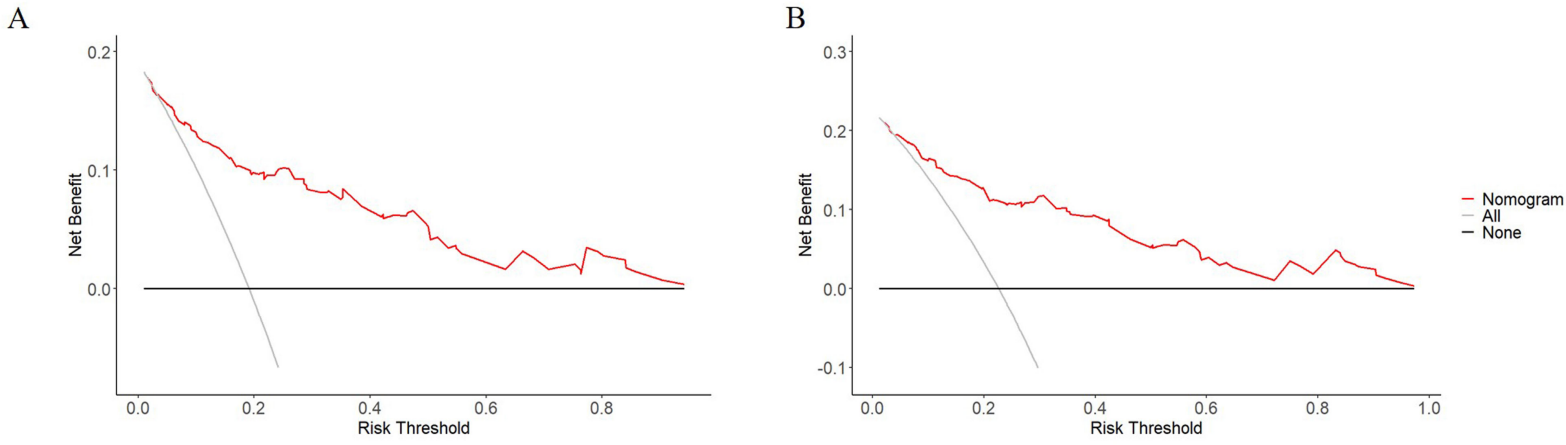
Decision curves analysis for the nomogram prediction model of overall survival in cervical cancer patients undergoing radiotherapy. **(A)** 3-year OS net benefit; **(B)** 5-year OS net benefit. The grey line represents the intervention for all patients, the black line represents no intervention for all patients, and the red line represents the net benefit of using the nomogram.

**Figure 6:**
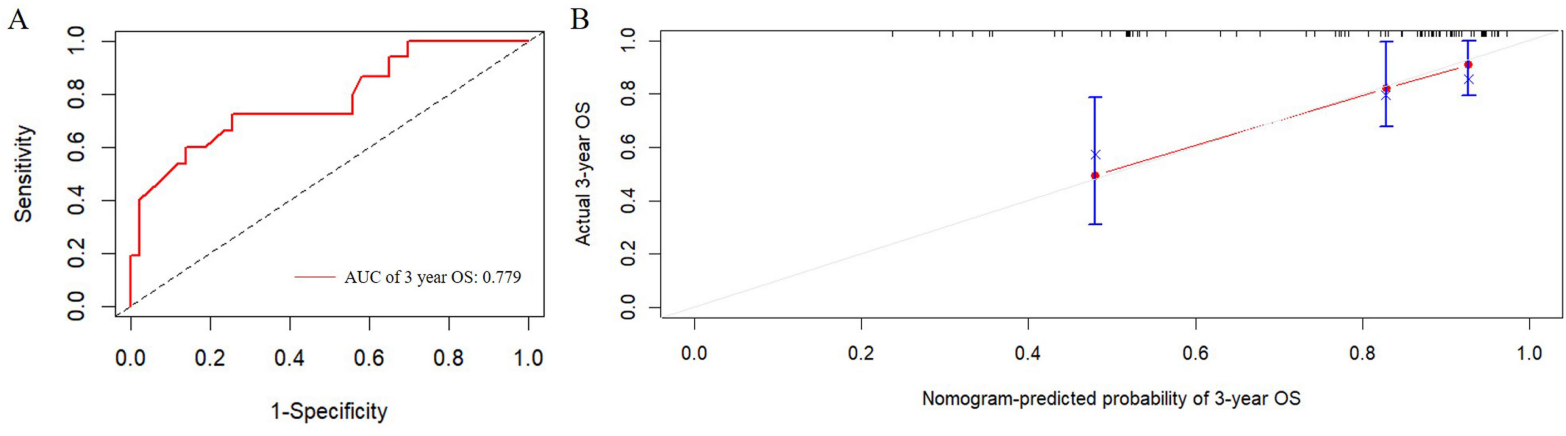
External validation of the predictive nomogram for patients with cervical cancer undergoing radiotherapy. **(A)** ROC curve of the 3-year OS. The AUC value of 3-year OS was 0.779 (95%CI: 0.635-0.922). **(B)** Calibration curve for predicting probability of the 3-year OS. ROC, receiver operating characteristic; AUC, area under the ROC curve.

**Figure 2** presented the nomogram prediction model for OS in cervical cancer patients undergoing radiotherapy, based on the results of the multivariate Cox regression analysis. The C-index of the nomogram model was 0.815 (95% CI: 0.766-0.864). The AUC values of 3-year and 5-year OS calculated by ROC curve were 0.849(95%CI: 0.789-0.909) and 0.840 (95%CI: 0.782-0.899), both greater than 0.8, indicating good discriminative ability (**Figure 3**). Furthermore, the calibration curves showed good agreement between predicted and actual probabilities, demonstrating high accuracy (**Figure 4**). The decision curve analysis (DCA) showed that the clinical net benefit of the model was higher than that of the extreme curves (None: no intervention for anyone; All: intervention for everyone) across most threshold probability ranges, indicating good clinical utility in most scenarios (**Figure 5**). The external validation results showed that the AUC value of 3-year OS by ROC curve was 0.779 (95%CI: 0.635-0.922), and the calibration curve showed that the model had good consistency, as shown in **Figure 6**.

## 4 Discussion

With the rapid development of medical imaging and computer technologies, precise radiotherapy techniques such as 3D-CRT and IMRT have become mainstream techniques in radiotherapy (21). Radiation enteritis is a common complication following radiotherapy for abdominal and pelvic malignancies. It is estimated that the probability of grade 2 or higher radiation enteritis in cervical cancer patients is approximately 15% to 30% (22, 23). This study analyzed data from 288 cervical cancer patients treated with either 3D-CRT or IMRT in our department, finding that 60 patients developed grade 2 or higher acute radiation enteritis, resulting in an incidence rate of 20.8%.

Multivariate Cox regression analysis in this study indicated that age ≥60 years, comorbidities, anemia, FIGO stage III-IV, poor differentiation, pelvic lymph node metastasis, NLR ≥2.54, and grade 2 or higher acute radiation enteritis were independent risk factors for OS in cervical cancer patients undergoing radiotherapy. Except for acute radiation enteritis, these significant factors have been previously reported as prognostic factors for cervical cancer patients (17–19, 23–25). The increased risk of mortality associated with age ≥60 years and comorbidities may be due to older cervical cancer patients typically presenting with more advanced disease and having a lower tolerance and enthusiasm for treatment. Anemia, as a critical factor, likely affects prognosis because it exacerbates hypoxia in tumor tissues, reducing the sensitivity of cancer cells to radiotherapy and chemotherapy, thus impairing treatment efficacy. Consistent with previous studies, later stages, lower differentiation, and pelvic lymph node metastasis were associated with poorer prognosis (17–19). It is well known that the prognosis of cervical cancer patients is affected by many factors, such as FIGO stage, LNM, comorbidity, etc, which may be more critical in evaluating the prognosis of cervical cancer patients. Although factors such as malnutrition and PLR have been confirmed by some studies to have clinical significance in the prognosis of cancer patients (15, 26, 27), they may change at any time due to clinical interventions such as nutritional support or pharmacotherapy. In addition, malnutrition and PLR may interact with other factors, as well as problems such as small sample size and heterogeneity of the included patients. All these most likely mask their direct relationship with the prognosis of cervical cancer patients in our study. The C-index and AUC values of the Nomogram prediction model based on independent risk factors in this study were greater than 0.8. and the calibration curve demonstrated good consistency between predicted and actual probabilities, indicating that the model had good discrimination ability and accuracy. The decision curve showed good clinical utility across most probability thresholds. Additionally, the external validation showed that the model had good clinical application value.

In recent years, the relationship between pre-treatment inflammatory parameters and prognosis of tumor patients has gradually attracted attention (10, 11). Inflammatory mediators in the tumor microenvironment may contribute to the proliferation and survival of malignant cells, promote tumor angiogenesis and metastasis, reduce the sensitivity of tumor to radiotherapy and chemotherapy, and ultimately affect the prognosis of patients (28). Domenici et al. reported that a higher pretreatment platelet count and PLR value may be used to predict poor prognosis in cervical cancer patients (29). Bruno et al. demonstrated that the median NLR and MLR of recurrent patients are higher, suggesting that they may be related to the recurrence of early-stage cervical cancer patients (30). For cervical cancer patients with stage IIB, both NLR and MLR are independent prognostic factors, and meanwhile NLR serves as a potential marker for therapeutic response (31). Studies have shown that NLR ≥ 3.1 is poor prognostic factors of nodal local control for cervical cancer patients (32). The inflammatory indicators have been proved as a tumor-specific prognostic indicator in many malignancies (33, 34), and our study also confirmed that cervical cancer patients with elevated NLR were associated with worse OS, which was consistent with the report by Han et al. (15, 35).

This study specifically analyzed the overall survival of patients with grade 0-1 versus grade 2 or higher acute radiation enteritis. Results showed that patients with grade 2 or higher acute radiation enteritis had significantly reduced overall survival, with 5-year OS rates of 79.2% and 70.0% for patients with grade 0-1 and grade 2 or higher acute radiation enteritis, respectively. The cause may be severe acute radiation enteritis, such as diarrhea, abdominal pain, bloody or mucous stool, which has a great impact on the quality of life of patients with cervical cancer, and even develops into chronic radiation enteritis. In addition, patients with grade 2 or higher radiation enteritis have serious intestinal injury, which affects the intestinal absorption capacity of patients with cervical cancer, may lead to malnutrition and even anemia, which has been confirmed to affect the prognosis of patients with cervical cancer (26, 27). These findings underscore the importance of preventing grade 2 or higher acute radiation enteritis in cervical cancer radiotherapy. Studies have shown that there are many factors affecting the development of acute radiation enteritis in patients with cervical cancer undergoing radiotherapy, such as the dose of radiation to the intestinal, concurrent chemotherapy, the type of radiotherapy technology (IMRT vs. CRT), the patient’s age, and nutritional status, etc (36–40). However, the clinical management strategy of acute radiation enteritis is mainly based on symptomatic treatment, including nutritional support, drug therapy, prevention and management of complications. Among them, drug therapy includes anti-inflammatory drugs, antibiotics, antioxidants, somatostatin therapy, etc. Surgical treatment is required when medical treatment is ineffective. Therefore, in clinical practice, measures should be taken to prevent and intervene in grade 2 or higher acute radiation enteritis, such as improving nutritional status, minimizing bowel dose during radiotherapy planning, and promptly managing acute radiation enteritis. However, as this study is a retrospective analysis, further multicenter, large-sample prospective studies are needed to confirm whether reducing the severity of acute radiation enteritis can improve the prognosis of cervical cancer patients undergoing radiotherapy.

In conclusion, the nomogram prediction model developed in this study can effectively predict the prognosis of cervical cancer patients undergoing radiotherapy. Grade 2 or higher acute radiation enteritis is an important prognostic factor for OS in these patients. Clinically, attention should be given to the prevention and intervention of acute radiation enteritis to improve patients’ quality of life and prognosis.

## Data Availability

The original contributions presented in the study are included in the article/supplementary material. Further inquiries can be directed to the corresponding author.
